# Mechanisms of Cytotoxicity of Chemical Agents to Giant Cell Tumors: An In Vitro Study

**DOI:** 10.1155/2020/8827192

**Published:** 2020-09-01

**Authors:** Achmad Fauzi Kamal, Akbar Rizki Beni Asdi, Ahmad Jabir Rahyussalim, Rio Wikanjaya, Resda Akhra Syahrani, Tri Kurniawati, Septelia Inawati Wanandi

**Affiliations:** ^1^Department of Orthopaedic and Traumatology, Faculty of Medicine Universitas Indonesia/Cipto Mangunkusumo General Hospital, Jakarta, Indonesia; ^2^Stem Cell Integrated Medical Technology Unit, Cipto Mangunkusumo General Hospital, Jakarta, Indonesia; ^3^Laboratorium of Stem Cell Research, Faculty of Medicine Universitas Indonesia, Jakarta, Indonesia; ^4^Department of Biochemistry and Molecular Biology, Faculty of Medicine Universitas Indonesia, Jakarta, Indonesia

## Abstract

**Background:**

Various chemical agents have been used as an adjuvant treatment for giant cell tumor (GCT). However, the comparative effect of these chemicals remains unclear.

**Methods:**

Multinucleated and spindle cells from cultured GCT patients, characterized by Nanog and Oct4 expression with RT-PCR, were directly administered, in vitro, with concentrations of 1%, 3%, and 5% of H_2_O_2_ and 75%, 85%, and 95% of ethanol for 10 minutes and concentrations of 0.003%, 0.005%, 0.01%, 0.03%, 0.1%, and 0.3% of H_2_O_2_ for 5 minutes and were incubated for 24 hours. Cell morphology, cell viability, and flow cytometry after various concentrations of H_2_O_2_ and ethanol exposure were assessed.

**Results:**

H_2_O_2_ in all concentrations caused loss of cell viability. The number of viable cells after H_2_O_2_ exposure was related to the concentration-dependent effect. The initial viable spindle-shaped cell, multinucleated giant cell, and round-epithelioid cell had morphological changes into fragmented nonviable cells after exposure to H_2_O_2_. Flow cytometry using Annexin V showed cell death due to necrosis, with the highest concentration amounting to 0.3%.

**Conclusion:**

Administering local chemical adjuvants of H_2_O_2_ in vitro caused loss of viable GCT cells. The number of viable cells after H_2_O_2_ exposure was related to the concentration-dependent effect, whereas reducing concentration of H_2_O_2_ may cause loss of viability and morphology of cultured GCT cells with the apoptotic mechanism.

## 1. Introduction

Giant cell tumor (GCT) of the bone is a benign tumor with the morphological findings of multinucleated giant cells and surrounding mononuclear stromal cells [1]. GCT is aggressive which has the potency to recur after simple intralesional excision (curettage) [2–5]. The incidence is 5% of all primary bone tumors and 20% of all benign bone tumors [6–8].

Curettage and reconstruction using cancellous bone graft to fill the defect is one of the treatments for controlling local tumor [9–11]. However, this treatment possesses a high recurrence rate of 25-50%. The use of local chemical adjuvants such as hydrogen peroxide (H_2_O_2_), phenol, ethanol, and liquid nitrogen after intralesional curettage showed the recurrence rate of 6-25%. This showed that the use of chemical adjuvants may increase the local control of the GCT of bone. Yet, agents with adequate effectivity and cytotoxicity were still unclear [12, 13].

We investigate the use of local chemical adjuvants (H_2_O_2_ and ethanol) in vitro on isolated osteoclast-like cells (multinucleated giant cells) in order to evaluate the most effective chemical adjuvant based on cell viability and cytotoxicity [14–16]. Studies comparing the effectivity and cytotoxicity of chemical adjuvants H_2_O_2_ and ethanol in isolated osteoclast-like cells (multinucleated giant cells) in vitro are limited. To date, there has been no research comparing the effectivity of chemical adjuvants in the treatment of GCT [17–20].

This was an experimental in vitro study, which isolated and cultured the GCT cells and administered the use of chemical agents H_2_O_2_ and ethanol. The result of this study has the aim of being able to aid in establishing the use of a chemical agent as an optimal local adjuvant in GCT cases, in order to effectively treat GCT and reduce its recurrence rate. Additionally, this study also shows the various methods available for isolation and culture of GCT.

## 2. Materials and Methods

This is an in vitro experimental study performed in the laboratory of Molecular Biology and Proteomics Core Facilities (MBPCF) IMERI of Faculty of Medicine Universitas Indonesia. The samples were obtained from GCT tissues of four patients in the operating room of our hospital. The diagnosis of GCT was made based on clinical, radiographic, and histopathological examination. Moreover, the diagnoses were discussed on the Clinicopathological Conference (CPC). Subjects had distal radius (two), proximal tibia (one), and proximal humerus (one) GCT, respectively. Inclusion criteria were patients with primary GCT in all types of bones, diagnosed based on CPC, first-time surgery, and willing to participate in the study. The exclusion criteria were recurrent GCT, damaged tumor tissue or failure to transport the tissue, and failure of a specimen to yield cells during culture. Informed consent and ethical approval were obtained before the tissue samples were obtained. Cell viability and cytotoxicity of chemical agents for each H_2_O_2_ and ethanol were assessed. All statistical analyses were performed using SPSS 24 software.

### 2.1. Isolation and Culture of GCT Cells (Multinucleated Giant Cells)

The tissue taken from the GCT primary tumor of the long bone was stored on a 50 mL Falcon tube (Biologix, China) in a 50 mL DMEM plain transport medium (Gibco, USA) with 5 mL FBS (Gibco, USA)+1.5 mL of penicillin/streptomycin (Gibco, USA)+1.5 mL amphotericin B (Gibco, USA)+0.3 mL gentamicin (Gibco, USA) at temperatures of 4°C until it was time to use. Then, the GCT tissue was placed in a Petri dish, followed by removing fat and necrotic tissue and cutting into small pieces. Furthermore, tissue was washed 5 times with 5 mL PBS (Life Technologies, USA) and 2% antibiotics using a 15 mL Falcon tube (Biologix, China). Fragments were placed into a new Petri dish, ensuring that there was no residual fat and blood. After that, it was cut into small fragments (±2-3 mm^3^) and divided into 2 for the explant method and collagenase method of cell culture [21–23].

In the explant method, 30 minutes of incubation of fragments in 1 mL PBS (Life Technologies, USA) on a 1.5 mL tube (Biologix, China) was performed using a 30-minute thermoshaker (SIA Biosan, Latvia) (37°C, 500 rpm). Then, we incised the base of 6 culture wells (Biologix, China) using a scalpel. The fragments were placed into the well by leaving it open for 15 minutes with the addition of the complete medium sufficiently about ±700 mL with 10% FBS (Gibco, USA), 1% penicillin/streptomycin (Gibco, USA), and amphotericin B (Gibco, USA) and stored in an incubator (Heracell, Thermo Scientific, USA) with CO_2_ of 5% and temperature of 37°C.

In the collagenase method, fragments are incubated in 1 mL of 0.4% collagenase IV (Northington, USA) in tube 15 (Biologix, China) and added with 10% PBS (Life Technologies, USA), penicillin 100 U/mL, and streptomycin 100 *μ*g/mL (Gibco, USA), by using a thermoshaker (3 hours, 37°C, and 500 rpm). After that, cell filtration was carried out, 1 mL of the complete medium was added for inactivation of collagenase IV, and then centrifugation (Biologix, China) at 1000 rpm for 10 minutes was performed. Then, the supernatant was removed with washers using 1 mL PBS (Life Technologies, USA) and centrifugation (Biologix, China) 1000 rpm for 10 minutes. After that, the supernatant was removed and resuspended in 1 mL complete DMEM+10% FBS (Gibco, USA). Fragments were placed in a 6-well culture plate (Biologix, China) and stored in an incubator (Heracell, Thermo Scientific, USA) with 5% CO_2_ and temperature of 37°C.

### 2.2. Subculture Characterization of GCT Stem Cells

If the culture had reached 90% confluence, the subculture/harvesting method was carried out in order to maintain nutrition and growth of GCT cells. Cell counting was performed using an automatic cell counter (Luna-II, Logos Biosystems, South Korea). The seeding process was performed by resuspending the HEK into the complete medium and adding 12-15 *μ*L cells into the suspension. Subsequently, cells were calculated under a microscope, and an amount of 5 × 10^4^ cells were taken and put on the well together with the medium and were incubated for 24 hours. PCR amplification, performed using SuperScript*®* III Reverse Transcriptase (Invitrogen, USA), was performed to see the expression of pluripotent gene markers, Nanog and Oct4 [24–26].

When the culture had reached 80% confluence containing mono- and multinuclear components, the culture was then treated with H_2_O_2_ and ethanol. The concentrations of H_2_O_2_ used were 1%, 3%, and 5% for 10 minutes and 0.0030%, 0.0050%, 0.01%, 0.03%, 0.1%, and 0.3% for 5 minutes, whereas concentrations of ethanol were 75%, 85%, and 95% for 10 minutes. The use of 1%, 3%, and 5% H_2_O_2_ was devised by Gortzak et al. [13] who mentioned it in their study, but the result of this study shows that higher concentration of H_2_O_2_ causes culture-wide cell death that leaves no viable tissue for analysis; hence, the culture was only treated with in vitro concentration (0.0030%, 0.0050%, 0.01%, 0.03%, 0.1%, and 0.3%) of H_2_O_2_ for 5 minutes.

### 2.3. Cell Morphology, Cell Viability, and Flow Cytometry Evaluations

After treatment with H_2_O_2_ and ethanol, cell morphology, cell viability, and flow cytometry were assessed to see the effect of the chemical substances. Ethical approval was obtained from the Ethical Committee of the Faculty of Medicine Universitas Indonesia/Cipto Mangunkusumo General Hospital.

**Figure 1:**
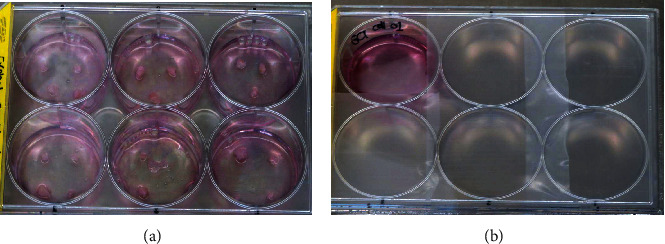
Isolation and culture of GCT of bone. (a) Explant method. (b) Collagenase method.

Cell culture was obtained from the tissues of 4 patients diagnosed with GCT of bone based on CPC. After the fat, blood, and necrotic tissues had been removed from the tumor, the tumor was cut into small pieces and divided into two groups, the collagenase and explant groups (Figure 1).

## 3. Results

### 3.1. Culture and Isolation of GCT Cells

**Figure 2:**
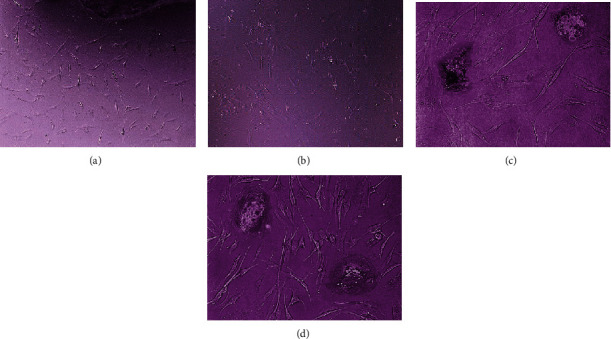
Morphological appearance of the cultured GCT cells. (a) Cell morphology of the explant method on day 7 (magnification 100x). (b) Cell morphology of the collagenase method on day 8 (magnification 100x). The cells of both methods were able to grow, producing the elongated, spindle-shaped, and round-epithelioid cells. (c) The cells of the explant method at week two (magnification 200x). (d) The cells of the collagenase method at week two (magnification 200x). The multinucleated giant cells along with the spindle-shaped and round-epithelioid cells appeared.

From those two methods of cell culture, all cells were able to grow at a mean day of 8. However, the explant method required less time (3 days faster) to grow the cells. Cells that were cultured using the explant method grew in the 5^th^ day. In the early days of growth, the majority of the cells were mononuclear cells with the morphology of elongated, spindle-shaped, and round-epithelioid cells. In subsequent days, multinucleated giant cells appeared (Figure 2). There was no difference in the morphology between identified samples in morphology.

### 3.2. Characterization of GCT Stem Cells

**Figure 3:**
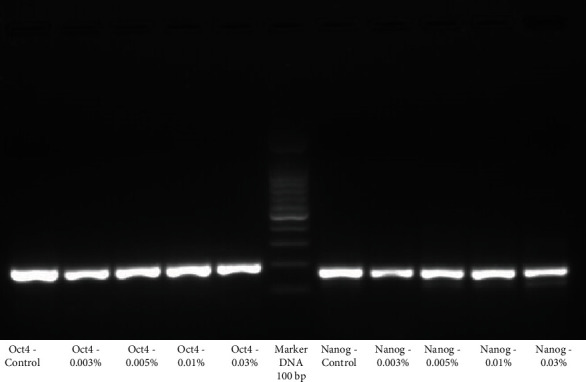
RT-PCR examination showed positive gene expression for the Nanog and Oct4 markers.

During RT-PCR examination, multinucleated giant cells, spindle-shaped mononuclear cells, and round-epithelioid cells showed positive gene expression for the Nanog and Oct4 markers. This indicated the stemness ability within the cells grown from our isolation and culture (Figure 3).

### 3.3. Cell Viability after Exposure to H_2_O_2_ and Ethanol

From our observation, H_2_O_2_ caused loss of cell viability in all concentrations. The GCT cell viability after administration of H_2_O_2_ concentrations of 1%, 3%, and 5% for 10 minutes showed significant differences in the number of viable cells when compared with controls (*p* values of 0.046, 0.043, and 0.043 for 1%, 3%, and 5% H_2_O_2_, respectively). It indicates that those concentrations of H_2_O_2_ more than 1% significantly caused loss of cell viability. The three “clinical concentrations” of H_2_O_2_ revealed comparable effectiveness for inhibition of GCT cell viability in vitro (*p* = 0.257).

**Figure 4:**
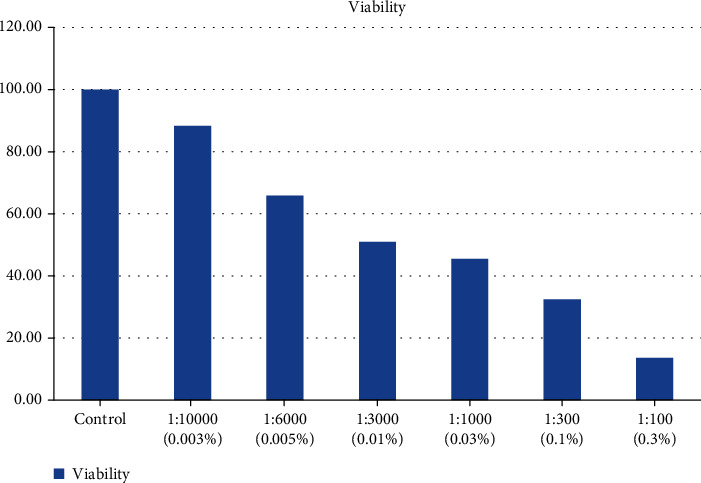
GCT cell viability after exposure to various in vitro concentrations of H_2_O_2_. The smallest number of viable cells of 13.65% was found after the administration of H_2_O_2_ concentration of 0.3%.

We found also significant differences between the treatment and control groups in the number of viable cells after exposure to in vitro concentrations of 0.0030%-0.3% for 5 minutes of H_2_O_2_ (*p* < 0.01). Among concentrations 0.01%, 0.03%, and 0.3% of H_2_O_2_, concentration 0.3% of H_2_O_2_ was the most effective one for inhibiting the viability of GCT cells in vitro (*p* = 0.027) (Figure 4).

Exposure of isolated and cultured GCT cells to 75%, 85%, and 95% ethanol caused the cell fixation phenomenon, where all cells stick to the bottom of the well; therefore, harvesting and analysis could not be performed.

### 3.4. Cell Morphology after Exposure to H_2_O_2_ and Ethanol

**Figure 5:**
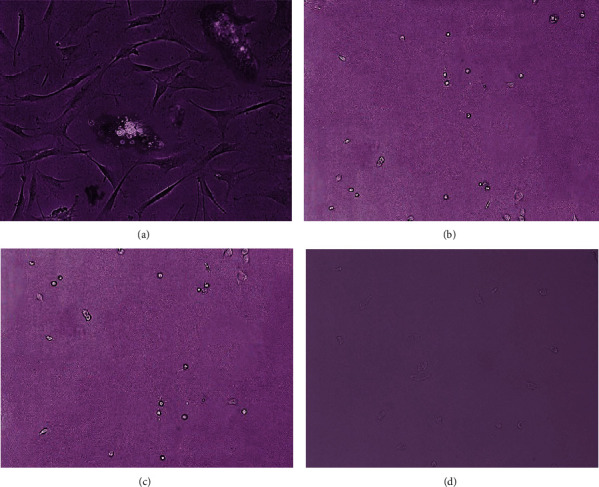
Comparison of cell morphology before and after exposure to H_2_O_2_ (magnification 200x). (a) Before exposure and after exposure to (b) 1%, (c) 3%, and (d) 5% H_2_O_2_, dead cell fragments which were released from the base of culture media.

After administration of concentrations of 1%, 3%, and 5% of H_2_O_2_ for 10 minutes, initial viable cells including the spindle-shaped cell, multinucleated giant cell, and round-epithelioid cell had morphological changes into fragmented nonviable cells (Figure 5).

**Figure 6:**
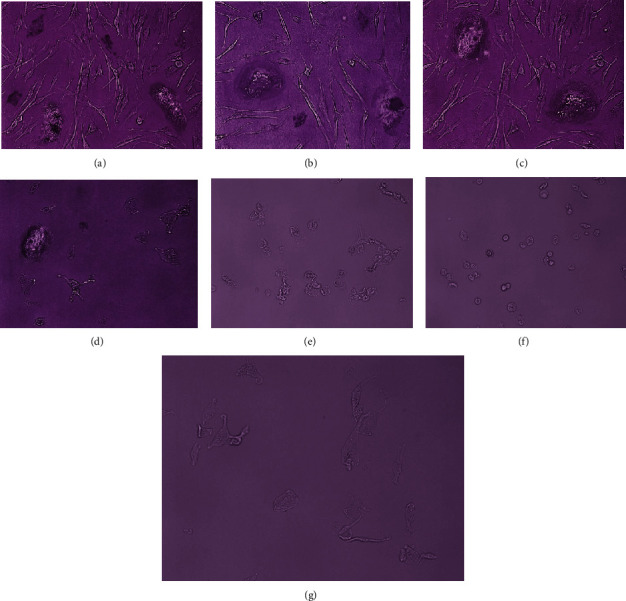
Comparison of cell morphology before and after exposure to H_2_O_2_ (magnification 200x). (a) Before exposure and after exposure to (b) 0.003%, (c) 0.005%, (d) 0.01%, (e) 0.03%, (f) 0.1%, and (g) 0.3% H_2_O_2_, the number of cells decreased with increasing concentration of H_2_O_2_ and multinucleated giant cells did not appear anymore with only a very small number of spindle-shaped cells left after exposure to H_2_O_2_ concentration of 0.3%.

Meanwhile, exposure to concentrations of 0.003%, 0.005%, 0.01%, 0.03%, 0.1%, and 0.3% H_2_O_2_ for 5 minutes also caused changes in morphology of cells and reduced the number of multinucleated giant cells and spindle-shaped cells. With increasing concentration given, multinucleated cells did not appear anymore and only a very small number of spindle-shaped cells left after exposure to H_2_O_2_ concentration of 0.3% (Figure 6).

### 3.5. GCT Stem Cells after Exposure to H_2_O_2_ and Ethanol

**Figure 7:**
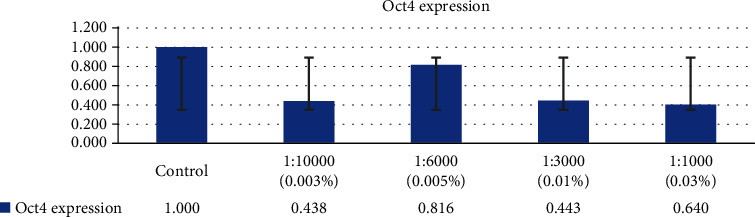
The expression of Oct4 from GCT stem cells decreased in line with increasing concentration of the H_2_O_2_.

**Figure 8:**
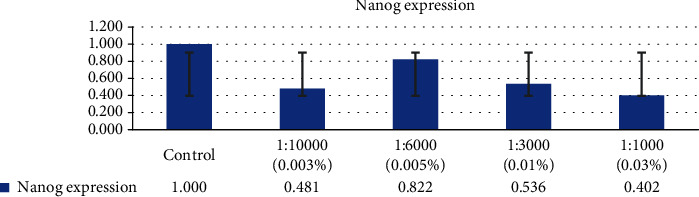
The expression of Nanog from GCT stem cells decreased in line with increasing concentration of the H_2_O_2_.

After exposure to in vitro concentration of H_2_O_2_, the measurement of Nanog and Oct4 was performed using RT-PCR with the result of the expression of Nanog and Oct4 from GCT stem cells decreased in line with the increasing concentration of the H_2_O_2_, as seen in Figures 7 and 8. It was found that after exposure to H_2_O_2_, the smallest expression of Nanog and Oct4 occurred in the H_2_O_2_ concentration of 0.03%.

### 3.6. Flow Cytometry Assessment

**Figure 9:**
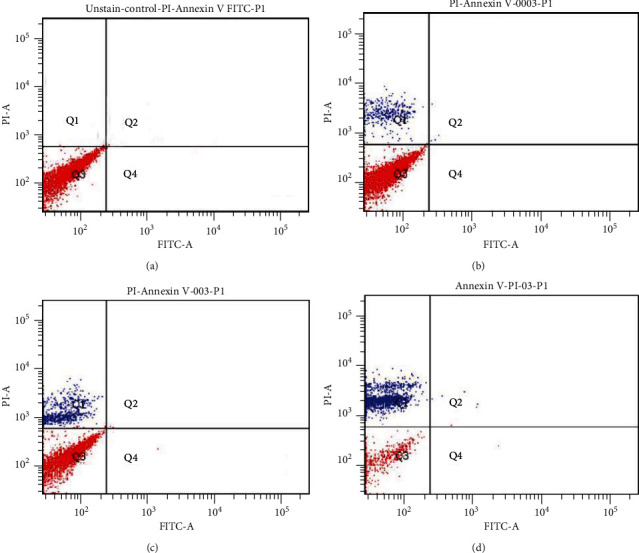
Plot graph examination of flow cytometry on markers of death of cells in the form of Annexin V and propidium iodide (PI). (a) Control group. (b) Group after administration of 0.003% H_2_O_2_. (c) Group after administration of 0.03% H_2_O_2_. (d) Group after administration of 0.3% H_2_O_2_.

The results of the flow cytometry examination were presented in a plot divided into 4 quadrants. The Q1 quadrant was for the propidium iodide- (PI-) FITC marker, the Q2 quadrant was for the PI-Annexin V FITC marker, the Q3 quadrant was for the baseline control, and the Q4 quadrant was for the Annexin V-FITC marker. From the plot in (Figure 9), it was concluded that the detection of the PI marker increased with the amount of H_2_O_2_concentration given. This indicates cell death that occurs after administration of H_2_O_2_ due to cell necrosis.

## 4. Discussion

Kamal et al. [27] stated that cell isolation and culture by the explant method produced more viability and final cell counts than the collagenase method. This was due to the fact that the explant method did not cause cell damage, whereas the enzymatic (collagenase) method may cause cell damage. Thus, the latter method might be a confounding factor. Hartley et al. [28] found no difference in the number of isolated cells of the two methods. Goldring et al. [29] concluded that the explant method produces cells that are purer and more homogeneous. In addition, enzymatic damage in such method is smaller than the collagenase one. Thus, the proliferation rate is higher compared to the collagenase method. Leung et al. [30] stated that tissue culture in the explant method took only one-third of the time required for the collagenase method and that cells obtained were 19 times higher than the collagenase method during primary culture. Our study showed that both of these methods were successful in growing the cells originating from GCT tissue with similar morphology. However, the explant method succeeded in growing cells faster with more viability and number of cells than the collagenase method.

Characterization of GCT stem cells was carried out by analyzing the RT-PCR pluripotent gene markers, Nanog and Oct4. Nanog expression is the characteristic of undifferentiated cancer cells, namely, CIS, embryonal carcinoma, and seminoma [27]. Besides Nanog, Oct4 is a gene specific to stem cells and was found to have upregulation on GCT [28]. In our study, we found that increased mRNA was detected in each culture on both transcription factors, where cell populations were able to express Nanog and Oct4 in RT-PCR analysis in all samples after exposure to H_2_O_2_. This postexposure RT-PCR examination is aimed at assessing the stemness ability among exposed GCT cells, which was found to decrease in a concentration-dependent manner. In another study conducted by Liu et al. [18], they concluded that the expression of Nanog and Oct4 on GCT cell culture on RT-PCR examination showed the characteristic of stemness of GCT cells.

This finding supported the research by Jain et al. [31], who proved the polyclonal nature of GCT cells in the way they carry out cell proliferation. This proves that tumor cells that divide in GCT come from one or more cells; this is usually found in neoplastic cells. Dhillon and Prasad [32] also succeeded in detecting GCT cell RNA through RT-PCR by finding the presence of estrogen receptors on their surface. In addition, to prove that cells isolated during culture had GCT stem cells, RT-PCR examination after administration of H_2_O_2_ showed a change in expression of these genes.

Based on the analysis of the various concentrations of H_2_O_2_ given, it was concluded that the concentration of 0.3% H_2_O_2_ decreased the expression of most genes. Bridge et al. [14], in their study, concluded that there was a significant decrease in the content of GCT cell DNA after exposure to the chemicals H_2_O_2_, phenol, ethanol, and ZnCl_2_.

Goldring et al. [29] found there are three types of cells during GCT culture. The first cell population is mononuclear cells with fibroblastic morphology which most likely represent tumor neoplastic elements, the second population is mononuclear cells that do not have receptors for skeletal hormones and do not survive in culture, and the third cell population consists of many nucleated giant cells that have nucleus-like mononuclear cells [29]. The cell population corresponds to what was found in this study. All cells isolated during culture had characteristics possessed by giant cell tumors; therefore, our study concluded that the explant and collagenase methods succeeded in isolating and culturing GCT cells from tumor tissue [30].

Our study demonstrated that administration of H_2_O_2_ with concentrations of 1%, 3%, and 5% for 10 minutes had the same effectiveness of loss of GCT cell viability in vitro. All cultured viable cells died after administration of these concentrations of H_2_O_2_. To perform morphological analysis, expression of Nanog and Oct4 genes and cytotoxicity tests require viable cells. Therefore, our study reduced H_2_O_2_ concentration and exposure time to 0.003%, 0.005%, 0.01%, 0.03%, 0.1%, and 0.3% by 5 minutes. We also found that exposure to concentrations 0.0030%-0.3% for 5 minutes of H_2_O_2_ causes the loss of viability of GCT cells. It could be concluded that the higher the concentration of H_2_O_2_, the higher the loss of viable cells in vitro. Our study revealed that the concentration 0.3% H_2_O_2_ for 5 minutes was the optimal concentration causing nonviable GCT cell culture. Riss et al. [33] performed an assessment of GCT metabolism after exposure to H_2_O_2_ with concentrations of 0.0034%, 0.034%, 0.01%, 0.34%, and 3.4%. The study concluded that a concentration of 3.4% resulted in lysis or death of all GCT cells resulting in decreased DNA content. They reported that cell damage was instant, substantial, and detected on examination under a microscope [30]. This is evidenced in our study with the therapy of H_2_O_2_; by giving increased concentration, the number of GCT cells decreased until the death of all cells.

The exposure to H_2_O_2_ concentrations of 1%, 3%, and 5% causes the death of all cells characterized by the loss of all spindle-shaped cell populations, multinucleated giant cells, and cells that detached from the surface of the culture (detachment) and left only formless cell fragments. The picture was in accordance with cell death due to necrosis. This is characterized by the destruction of the plasma membrane so that the remaining visible pieces were only cell fragments. The exposure to H_2_O_2_ concentrations below 1% caused cell morphological changes and reduced the number of multinucleated giant cells and spindle-shaped cells. Loss of cell viability and morphological changes after H_2_O_2_ exposure may be caused by damage to cell membranes, deactivate enzymes, and damage DNA [34]. This was in line with the research studies by Gortzak et al. [13], López-Lázaro [35], and Jain et al. [31].

Cell exposure to ethanol concentrations of 75%, 85%, and 95% could not be analyzed. This was due to the phenomenon of cell fixation in the form of denaturation due to 10 minutes of ethanol exposure in all groups. Fixation resulted in the deposition of cells on the basis of well culture media, so that cells could not be investigated and analyzed. This was in accordance with the nature of ethanol as a fixative agent in tissue preparation [32].

Annexin V is a phospholipid-binding protein that can bind phosphatidylserine (PS) [36]. PS is normally located on the inside of living cell plasma membranes but can be found on the cell surface that undergoes apoptosis and serves as an identification signal in eliminating apoptotic cells by macrophages [33].

The principle of flow cytometry in cell death detection is to record the appearance of cells at the peak of G0/G1 observed after cells were given a specific DNA staining in the form of Annexin V for apoptosis and PI for necrosis. This process results from the degradation product chromatin from cells, the formation of the apoptotic body (the fragmentation of dying cells and its nucleus), and the extraction of chromatin degradation products from cell drying during cell preparation and staining [37].

Cells that experience necrosis will be detected by a PI marker. PI is a fluorescent dye, which cannot pass through cell membranes, but binds DNA and RNA. Bonding of PI with RNA and DNA indicates damage to cell membranes so that dead cells are recorded in flow cytometry [13]. Examination of flow cytometry produces a plot with a picture of points scattered in 4 quadrants. The lower left quadrant shows cells that are still viable as the baseline, and the other quadrants represent dead cells due to the mechanism of necrosis with PI markers (top left), apoptosis with Annexin V markers (lower right), or combinations (top right). Our study showed that cell death after administration of chemical adjuvants was due to necrosis, characterized by the detection of dead cells in PI markers in the upper left quadrant. The number of necrotizing GCT cells increases with increasing H_2_O_2_ concentration. Between 0.0030% and 0.3% concentrations of H_2_O_2_, it was concluded that 0.3% of H_2_O_2_ was the optimal concentration for 80% of necrosis of cultured GCT cells.

Our study had the same result as the study by Verdegaal et al. [38] in which they had assessed the mechanism of chondrosarcoma cancer cell death in flow cytometry. They concluded that cell death after administration of H_2_O_2_ in vitro was due to necrosis. Our study was in accordance with Gortzak et al. [13] and Nicholson et al. [34], who concluded that cell death after exposure to H_2_O_2_ was due to necrosis indicated by destruction of the cell plasma membrane leaving only cell fragments. This death by means of necrosis supported the findings of previous morphological images: cells that have been exposed to H_2_O_2_ undergo lysis and damage to cell membranes. In contrast to the administration of bortezomib agents in a study by Bao et al. [39], they stated that cell death occurs due to apoptosis; this is indicated by the presence of cells in the Annexin marker in the examination of flow cytometry.

## 5. Conclusion

Isolation and cell culture from GCT tissue could be done by the collagenase and explant methods. Both of these methods were able to grow cells from GCT tissue with similar morphology. However, the explant method produced more cells in less time compared to the collagenase method. Cells grown in culture contained GCT stem cells as evidenced by the expression of the Nanog and Oct4 markers.

H_2_O_2_ caused loss of cell viability in all concentrations. We found a concentration-dependent effect of H_2_O_2_: increased H_2_O_2_ concentration was correlated with decreased expression of both Nanog and Oct4 genes.

We concluded also that the concentrations of H_2_O_2_ more than 1% significantly caused the loss of cell viability, supported by morphological changes from the initial viable spindle-shaped cell, multinucleated giant cell, and round-epithelioid cell into fragmented nonviable cells after exposure to H_2_O_2_. Reducing concentration of H_2_O_2_ also caused loss of the number of viable GCT cells with apoptosis.

One of our limitations was that, in this study, GCT cells that were killed by ethanol could not be harvested as the cells were fixed. Moreover, as this was an in vitro study, animal studies are required to investigate the safety of H_2_O_2_ for treating GCT.

## Data Availability

The data used to support the findings of this study are available from the corresponding author upon request.

## References

[1] McGrath P. J. (1972). Giant-cell tumour of bone. *The Journal of Bone and Joint Surgery British Volume*.

[2] Trieb K., Bitzan P., Lang S., Dominkus M., Kotz R. (2001). Recurrence of curetted and bone-grafted giant-cell tumours with and without adjuvant phenol therapy. *European Journal of Surgical Oncology*.

[3] Wülling M., Delling G., Kaiser E. (2003). The origin of the neoplastic stromal cell in giant cell tumor of bone. *Human Pathology*.

[4] Wülling M., Engels C., Jesse N., Werner M., Delling G., Kaiser E. (2001). The nature of giant cell tumor of bone. *Journal of Cancer Research and Clinical Oncology*.

[5] Prosser G. H., Baloch K. G., Tillman R. M., Carter S. R., Grimer R. J. (2005). Does curettage without adjuvant therapy provide low recurrence rates in giant-cell tumors of bone?. *Clinical Orthopaedics and Related Research*.

[6] Chakarun C. J., Forrester D. M., Gottsegen C. J., Patel D. B., White E. A., Matcuk G. R. (2013). Giant cell tumor of bone: review, mimics, and new developments in treatment. *Radiographics*.

[7] Georgiev G. P., Slavchev S., Dimitrova I., Landzhov B. (2014). Giant cell tumor of bone: current review of morphological, clinical, radiological, and therapeutic characteristics. *Journal of Clinical and Experimental Investigations*.

[8] López-Pousa A., Broto J. M., Garrido T., Vázquez J. (2015). Giant cell tumour of bone: new treatments in development. *Clinical & Translational Oncology*.

[9] Puri A., Agarwal M. G., Shah M., Jambhekar N. A., Anchan C., Behle S. (2007). Giant cell tumor of bone in children and adolescents. *Journal of Pediatric Orthopedics*.

[10] Shih H. N., Hsu R. W. W., Sim F. H. (1998). Excision curettage and allografting of giant cell tumor. *World Journal of Surgery*.

[11] Prabowo Y., Abubakar I. (2018). Reconstruction giant cell tumor of the right proximal humerus Campanacci 3 with pedicle and rod system: a case report. *International Journal of Surgery Case Reports*.

[12] Sobti A., Agrawal P., Agarwala S., Agarwal M. (2016). Giant cell tumor of bone - an overview. *Archives of Bone and Joint Surgery*.

[13] Gortzak Y., Kandel R., Deheshi B. (2010). The efficacy of chemical adjuvants on giant-cell tumour of bone. *The Journal of Bone and Joint Surgery British Volume*.

[14] Bridge J. A., Neff J. R., Mouron B. J. (1992). Giant cell tumor of bone. Chromosomal analysis of 48 specimens and review of the literature. *Cancer Genetics and Cytogenetics*.

[15] Kamal A. F., Waryudi A., Effendi Z., Kodrat E. (2016). Management of aggressive giant cell tumor of calcaneal bone: a case report. *International Journal of Surgery Case Reports*.

[16] Kamal A. F. (2015). Sacral tumor: experience in a single institution. *Indonesian Journal of Cancer*.

[17] Zhou Z., Li Y., Wang X. (2018). ALCAM^+^ stromal cells: role in giant cell tumor of bone progression. *Cell Death & Disease*.

[18] Liu L., Aleksandrowicz E., Fan P. (2014). Enrichment of c-Met^+^ tumorigenic stromal cells of giant cell tumor of bone and targeting by cabozantinib. *Cell Death & Disease*.

[19] Kim Y., Nizami S., Goto H., Lee F. Y. (2012). Modern interpretation of giant cell tumor of bone: predominantly osteoclastogenic stromal tumor. *Clinics in Orthopedic Surgery*.

[20] Robinson D., Segal M., Nevo Z. (2002). Giant cell tumor of bone. *Pathobiology*.

[21] Littlewood-Evans A., Kokubo T., Ishibashi O. (1997). Localization of cathepsin K in human osteoclasts by in situ hybridization and immunohistochemistry. *Bone*.

[22] Schajowicz F. (1961). Giant-cell tumors of bone (osteoclastoma). *The Journal of Bone & Joint Surgery*.

[23] Murphey M. D., Nomikos G. C., Flemming D. J., Gannon F. H., Temple H. T., Kransdorf M. J. (2001). From the archives of AFIP. Imaging of giant cell tumor and giant cell reparative granuloma of bone: radiologic-pathologic correlation. *Radiographics*.

[24] Georgiev G., Georgiev H., Landzhov B. (2012). Ultrastructural study of giant cell tumor of bone. *Orthop. Rheumatol J*.

[25] Georgiev G. P., Landzhov B., Slavchev S. A. (2015). Comparative electron microscopic and immunohistochemical study of stromal cells in giant cell tumor of bone. *Scripta Scientifica Medica*.

[26] Zheng M. H., Robbins P., Xu J., Huang L., Wood D. J., Papadimitriou J. M. (2001). The histogenesis of giant cell tumour of bone: a model of interaction between neoplastic cells and osteoclasts. *Histology and Histopathology*.

[27] Kamal A. F., Pranatha D. Y., Sugito W., Rahman F., Susanto E. (2018). Culture and Characterization of Cancer Stem Cells from Primary Osteosarcoma. *The Open Stem Cell Journal*.

[28] Hart A. H., Hartley L., Parker K. (2005). The pluripotency homeobox gene *NANOG* is expressed in human germ cell tumors. *Cancer*.

[29] Goldring S. R., Roelke M. S., Petrison K. K., Bhan A. K. (1987). Human giant cell tumors of bone. Identification and characterization of cell types. *The Journal of Clinical Investigation*.

[30] Leung K.-S., Huang L., Qin L., Qin Y.-X., Cheung W.-H., Zheng M.-H. (2010). Tissue culture of giant cell tumor of bone. *A Practical Manual for Musculoskeletal Research 2008*.

[31] Jain R., Kumar S., Gupta A., Arunim S., Shrimal R. (2017). Efficacy of hydrogen peroxide in preventing recurrence of giant cell tumor of the bone. *Journal of Bone and Joint Diseases*.

[32] Dhillon M. S., Prasad P. (2007). Multicentric giant cell tumour of bone. *Acta Orthopaedica Belgica*.

[33] Riss T., Moravec R., Niles A., Duellman S., Beningk H., Worzella T. (2004). *Assay Guidance Manuals 2004*.

[34] Nicholson N. C., Ramp W. K., Kneisl J. S., Kaysinger K. K. (1998). Hydrogen peroxide inhibits giant cell tumor and osteoblast metabolism in vitro. *Clinical Orthopaedics and Related Research*.

[35] López-Lázaro M. (2007). Dual role of hydrogen peroxide in cancer: possible relevance to cancer chemoprevention and therapy. *Cancer Letters*.

[36] Borowski M., Giovino-Doherty M., Ji L., Shi M., Smith K., Laning J. (2012). *The Stem Cell Research Community*.

[37] Jurišić V., Bumbaširević V. (2008). In vitro assays for cell death determination. *Archive of Oncology*.

[38] Verdegaal S. H. M., Corver W. E., Hogendoorn P. C. W., Taminiau A. H. M. (2008). The cytotoxic effect of phenol and ethanol on the chondrosarcoma-derived cell line OUMS-27. *The Journal of Bone and Joint Surgery British Volume*.

[39] Bao X., Ren T., Huang Y. (2017). Bortezomib induces apoptosis and suppresses cell growth and metastasis by inactivation of Stat3 signaling in chondrosarcoma. *International Journal of Oncology*.

